# NAF-1 Inhibition by Resveratrol Suppresses Cancer Stem Cell-Like Properties and the Invasion of Pancreatic Cancer

**DOI:** 10.3389/fonc.2020.01038

**Published:** 2020-07-16

**Authors:** Tao Qin, Liang Cheng, Ying Xiao, Weikun Qian, Jie Li, Zheng Wu, Zheng Wang, Qinhong Xu, Wanxing Duan, Lucas Wong, Erxi Wu, Qingyong Ma, Jiguang Ma

**Affiliations:** ^1^Department of Hepatobiliary Surgery, The First Affiliated Hospital of Xi'an Jiaotong University, Xi'an, China; ^2^Department of Oncology, Baylor Scott & White Health, Temple, TX, United States; ^3^Department of Surgery, Texas A&M University College of Medicine, Temple, TX, United States; ^4^Department of Neurosurgery, Baylor Scott & White Health, Temple, TX, United States; ^5^Neuroscience Institute, Baylor Scott & White Health, Temple, TX, United States; ^6^Department of Pharmaceutical Sciences, Texas A&M University College of Pharmacy, College Station, TX, United States; ^7^LIVESTRONG Cancer Institutes, Dell Medical School, The University of Texas at Austin, Austin, TX, United States; ^8^Department of Oncology, Dell Medical School, The University of Texas at Austin, Austin, TX, United States; ^9^Department of Anesthesiology, The First Affiliated Hospital of Xi’an Jiaotong University, Xi’an, China

**Keywords:** resveratrol, NAF-1, cancer stem cells, pancreatic cancer, progression

## Abstract

Resveratrol is a natural polyphenolic compound with multiple biological effects, *e.g*., proliferation inhibition, anti-oxidation, and neuroprotection. Besides that, studies have shown that resveratrol inhibits tumor growth and migration, as well as epithelial–mesenchymal transition (EMT). However, its molecular mechanisms in tumor progression are not fully understood. Nutrient-deprivation autophagy factor-1 (NAF-1) is mainly found in the endoplasmic reticulum and mitochondrial outer membrane. It is an important genetic locus for regulating oxidative stress and autophagy. The molecular mechanism of NAF-1 in pancreatic cancer is currently unclear. The current study found that NAF-1 is expressed in pancreatic cancer tissue and correlated with the progression of pancreatic cancer. Furthermore, we found that NAF-1 inhibition significantly inhibits the stem cell characteristics and the invasion and migration abilities of pancreatic cancer cells. In a subcutaneous xenograft model of pancreatic cancer in nude mice, resveratrol inhibited the expression of NAF-1, thereby inhibiting tumor growth. Taken together, resveratrol could be an effective anti-tumor drug, and NAF-1 may be a rational therapeutic target.

## Introduction

Pancreatic cancer is one of the deadly malignant tumors, with a 5-year survival rate of <9% (1). For patients who cannot undergo an operation, chemotherapy is the only standard treatment option (2). However, due to the biological characteristics of pancreatic cancer cells, they are insensitive to a variety of chemotherapeutic drugs and show multidrug resistance. In the context of the increasing incidence each year, deepening our understanding of pancreatic cancer and the mechanism of chemoresistance in pancreatic cancer is of great significance for improving the effect of chemotherapy.

Resveratrol has a variety of biological effects, such as anti-oxidant, anti-inflammatory, cardioprotective, neuroprotective, and anti-diabetic effects (3). In recent years, many studies have shown that resveratrol can directly inhibit the proliferation of tumor cells and induce growth inhibition, cell cycle arrest, and apoptosis in pancreatic cancer. As a result, the progression of the tumor is prevented by resveratrol (4). However, the specific molecular mechanism of resveratrol in the treatment of cancer is still unclear, and further research is needed.

Cancer stem cells (CSCs) are defined as cells within the tumor that have the ability to self-renew, which allows these cells to develop into different types of cancer cell lines (5), forming new tumors and subsequently causing recurrence and metastasis. Therefore, the activity of CSCs leads to treatment failure. CSCs play an important role in the progression of cancer, such as the occurrence, maintenance, invasion, immunosuppression, and drug resistance of tumors (6). Pancreatic cancer stem cells (PCSCs) have been reported to be involved in a variety of signaling pathways, including the Notch, Hedgehog, Wnt/ß-catenin, JNK, and c-Met pathways (7). Shen et al. (8) showed that resveratrol has the ability to reverse epithelial–mesenchymal transition (EMT), impair the initiation of stem cell-like properties in tumors, inhibit proliferation, and enhance the radiosensitivity of cancer. In human PCSCs and in PCSCs produced by KRAS^GD12^ transgenic mice, Shankar et al. (9) also conducted experimental studies to demonstrate that resveratrol inhibits the properties of certain PCSCs by inhibiting various cytokines, drug-resistant genes, and EMT. Furthermore, resveratrol induced tumor cell apoptosis by activating capase-3/7 and inhibiting bcl-2 and XIAP in pancreatic cancer.

Nutrient-deprivation autophagy factor 1 (NAF-1), a nutrient deficiency-induced autophagy factor, mainly exists in the endoplasmic reticulum and mitochondrial outer membrane (10). Studies have found that NAF-1 is mainly involved in the regulation of oxidative stress and autophagy and is closely related to tumor growth (11). The high expression of NAF-1 indicated poor prognosis. *In vivo* and *in vitro* experiments also confirmed that NAF-1 can promote the proliferation and the tumorigenicity of tumor cells. At present, the molecular mechanism of NAF-1 in tumors, especially in pancreatic cancer, is still unclear.

In the present study, we sought to explore the role of NAF-1 and resveratrol in stem cell characteristics and to explore their contribution to the progression of pancreatic cancer.

## Materials and Methods

All experimental protocols were approved by the Ethical Committee of the First Affiliated Hospital, Xi'an Jiaotong University, Xi' an, China.

### Reagents and Antibodies

Resveratrol (>99% pure) was purchased from Sigma-Aldrich (St. Louis, MO, USA), was dissolved in dimethyl sulfoxide (DMSO) to prepare 50- and 10-mM stock solutions and stored at −20°C. DMSO was used as the vehicle control.

Primary antibodies against SOX2 (No. ab93689, 1:1,000), NANOG (No. ab109250, 1:1,000), and OCT4 (No. ab18976, 1:1,000) were purchased from Abcam (Cambridge, MA, USA). N-cadherin (No. 13116S, 1:1,000), E-cadherin (No. 3195S, 1:1,000), and Vimentin (No. 5741, 1:1000) were provided by Cell Signaling Technology (Danvers, MA, USA). The secondary antibodies [anti-rabbit IgG (No. ab6721, 1:10,000) and anti-mouse IgG (No. ab6728, 1:10,000)] were provided by Abcam (Cambridge, MA, USA). The antibodies against NAF-1 (No. 13318-1-AP, 1:1000), α-tubulin (No. 66031-1-Ig, 1:5,000) were purchased from Proteintech Group (Chicago, IL, USA). Other reagents were purchased from common commercial sources.

### Human Tissue Specimens and Histological Analyses

Pancreatic cancer tissues (91 cases) were collected from the Department of Hepatobiliary Surgery, and normal pancreatic tissue (five cases) were obtained from patients undergoing liver transplantation at the First Affiliated Hospital of Xi'an Jiaotong University. The sixth edition of the TNM classification of the American Joint Commission on Cancer was used to assess the pathological TNM status in this study. Two pathologists examined the pathological factors. The clinical pathological data are summarized in Table 1. Immunohistochemistry was performed according to the methods described in a previous study (12).

**Table 1:** Statistical relationship between the expression of NAF-1 and the clinicopathological features in 91 cases of pancreatic cancer.

	**Samples**	**NAF-1 expression**, ***n*** **(%)**				
	**(*n*)**	**None**	**Weak**	**Moderate**	**Strong**	***p*-value^◇^**
Sex						0.478
Male	59	5 (8.5)	13 (22.0)	35 (59.3)	6 (10.2)	
Female	32	4 (12.5)	11 (34.4)	14 (43.8)	3 (9.4)	
Age						0.312
≤ 51.4^a^	45	2 (4.4)	14 (31.1)	24 (53.3)	5 (11.1)	
>51.4	46	7 (15.2)	10 (21.7)	25 (54.3)	4 (8.7)	
Histological grade						0.435
1	26	2 (7.7)	5 (19.2)	16 (61.5)	3 (11.5)	
2	41	6 (14.6)	14 (34.1)	17 (41.5)	4 (9.8)	
3	24	1 (4.2)	5 (20.8)	16 (66.7)	2 (8.3)	
TNM stage						0.002
(American Joint Commission on Cancer)						
I	16	2 (12.5)	2 (12.5)	10 (62.5)	2 (12.5)	
II	60	5 (8.3)	20 (33.3)	34 (56.7)	1 (1.7)	
III	9	2 (22.2)	1 (11.1)	2 (22.2)	4 (44.4)	
IV	6	0 (0)	1 (16.7)	3 (50.0)	2 (33.3)	
pT status						0.033
T1	2	1 (50.0)	0 (0)	1 (50.0)	0 (0)	
T2	14	5 (35.7)	2 (14.3)	6 (42.9)	1 (7.1)	
T3	66	3 (4.5)	20 (30.3)	36 (54.5)	7 (10.6)	
T4	9	0 (0)	2 (22.2)	6 (66.7)	1 (11.1)	
pN status						0.275
N0	71	6 (8.5)	22 (31.0)	36 (50.7)	7 (1.4)	
N1	20	3 (15.0)	2 (10.0)	13 (65.0)	2 (10.0)	
pM status						0.217
M0	85	9 (10.6)	23 (27.1)	46 (54.1)	7 (8.2)	
M1	6	0 (0)	1 (16.7)	3 (50.0)	2 (33.3)	

◇ , x^2^ test; *p < 0.05 (significantly different).

a *mean age*.

NAF-1 staining status was evaluated according to cytoplasmic expression. NAF-1 staining was classified into the following four groups based on the percentage of positive cells and the staining intensity: negative (0), weak (1+), moderate (2+), and strong (3+). Specifically, based on the percentage of positive cells (percentage scores), the samples were scored as follows: <10% (0), 10–25% (1), 25–50% (2), 50–75% (3), and >75% (4). The intensity of staining was scored with the following four grades (intensity scores): no staining (0), light brown (1), brown (2), and dark brown (3). NAF-1 staining status was determined by the following formula: overall score = percentage score × intensity score. An overall score of ≤ 3 was defined as negative (0), an overall score > 3 and ≤ 6 was defined as weak (1+), an overall score > 6 and ≤ 9 was defined as moderate (2+), and an overall score of > 9 was defined as strong (3+).

### Cell Lines and Cell Culture

The human Panc-1, MiaPaCa-2, BxPC-3, CF PAC-1, and SW1990 pancreatic cancer cells were used in this study, and they were purchased from the Cell Bank of Type Culture Collection of the Chinese Academy of Sciences (Shanghai, China). All cells were cultured in proper media (HyClone, Logan, USA), and 10% fetal bovine serum (FBS) and 1% penicillin and streptomycin were added into the proper media. A humidified atmosphere at 37°C with 5% CO_2_ was provided to culture all cells. Pancreatic stellate cells (PSC) were isolated from the tissue and cultured as previously described (13). The study was conducted following the Declaration of Helsinki, and all protocols were approved by the relevant ethical committee of the First Affiliated Hospital of Xi'an Jiaotong University, Xi'an, China.

### Stable Lentiviral Transfection

NAF-1 shRNA (shNAF-1) and negative control shRNA (shNC), which were in eukaryotic GV248 lentiviral vectors, were used to establish stably transfected cells. The eukaryotic GV248 lentiviral vectors were used to establish the stable NAF-1-overexpression cells. All transfection reagents and materials were purchased from GeneChem Co., Ltd. (Shanghai, China).

Before transfection, the cells were seeded into six-well plates at 1 × 105 cells/well. Polybrene (5 μg/ml) and ENi.S (reagent for enhancing the effect of lentivirus transfection) were used during virus transfection according to the manufacturer's protocol. Transfection was carried out using lentiviral particles [Panc-1, multiplicity of infection (MOI) = 10; BxPC-3, MOI = 20]. At 12 h after transfection, a virus-containing medium was replaced with the fresh medium. Puromycin (Merck, USA) was used to select the successfully transfected cells when it was 96 h after transfection.

The media was changed three times per week after transfection to generate stably transfected cells. Puromycin-resistant colonies were isolated after 3 weeks. The stable NAF-1-suppressed/NAF-1-overexpressed pancreatic cancer cells were named shNAF-1/NAF-1(+) and the control pancreatic cancer cells were named shNC. qRT-PCR (not shown) and Western blot were used to analyze the effect of gene silencing and the effect of gene over-expression.

### Western Blot Analysis

After washing with phosphate-buffered saline (PBS) and according to the manufacturer's instructions, the pancreatic cancer cells (Panc-1 and MiaPaCa-2 cells) were lysed by RIPA buffer (Beyotime, Guangzhou, China). Whole-cell lysates were collected for the subsequent experiments. Bicinchoninic acid protein assay kit (Pierce, Rockford, USA) was used to determine the protein concentration.

Next, 10% polyacrylamide gel, with 5% stacking gel, was used to subject the proteins to sodium dodecyl sulfate-polyacrylamide gel electroporesis. Then, the proteins were transferred to polyvinylidene difluoride (PVDF) membranes. Fat-free milk (10%) which was dissolved with Tris-buffered saline-Tween (TBST) was used to block the PVDF membranes for 2 h at room temperature. Then, all the membranes were incubated with primary antibodies at 4°C overnight. After washing three times with TBST, secondary horseradish peroxidase-conjugated antibodies were used to incubate the membranes for 2 h at room temperature. An enhanced chemiluminescence kit and the Molecular Imager ChemiDoc XRS System (Bio-Rad, Hercules, CA, USA) were used to detect immunocomplexes after washing three times with TBST.

### Immunofluorescence Staining

Paraformaldehyde (4%) was used to fix the cells for 15 min. After washing in PBS, 0.3% Triton X-100 was used to permeabilize the cells. Bovine serum albumin (5%), in PBS, was used to block the cells for 30 min at room temperature. Then, primary antibodies were used to incubate the cells overnight at 4°C. After washing three times with PBS, fluorescently labeled secondary antibodies were used to incubate the cells for 2 h at room temperature. 4′,6-Diamidino-2-phenylindole was used to stain the nuclei for 1 min at room temperature. Finally, Zeiss Instruments confocal microscope was used to detect the fluorescence.

### Spheroid Formation Assay

We have fully resuspended all the pancreatic cancer cells including Panc-1 and BxPC-3 until they separated into single cells (observed through a microscope) before planting. Panc-1 and BxPC-3 (1 × 10^4^ cells/well) were cultured in six-well ultralow attachment plates (Corning, Corning, NY, USA) in Dulbecco's Modified Eagle's Medium (DMEM) containing EGF, bFGF, and B27 and grown at 37°C under 5% humidified CO_2_-containing atmosphere for 2 weeks. The number of tumor spheres was counted with a microscope (Nikon Instruments Inc.).

### Cell Invasion Assay

Before inoculating the cells, Transwell chambers were placed in a 24-well plate, and diluted Matrigel matrix gel (1:8) was added. In addition to the 24-well plate, the prepared Transwell chambers were kept in an incubator at 37°C overnight. Before using the chambers, 50 μl of serum-free DMEM culture solution was added to each chamber membrane to hydrate the substrate. A total of 700 μl of complete culture medium, containing 10% FBS, was added to the 24-well plate, and 200 μl of preintervention and diluted cell suspension was inoculated into the Transwell chambers. After 24–48 h of incubation in a constant-temperature incubator at 37°C with 5% CO_2_, the Transwell chambers were fixed with 4% paraformaldehyde for 2 min. Then, methanol was added, and the solution was mixed for 20 min. Next, the cells in the chamber were stained with 0.3% crystal violet dye for 15 min. Finally, cell images were observed with an inverted microscope and recorded with a camera.

### The Scratch Assay

After digestion, the cells in the different groups were uniformly inoculated into six-well plates, and the density reached approximately 5 × 10^5^ cells/ml. Panc-1 and BxPC-3 cells were cultured at 37°C with 5% CO_2_ and grown in a single layer. When confluency reached 100%, a sterile ruler was used to draw straight lines. Then, PBS was used to remove the suspended cells, and serum-free medium was added. The results were observed and recorded with an inverted microscope (Nikon Corporation) at 0, 24, and 48 h after the six-well plates were kept at 37°C with 5% CO_2_.

### *In vivo* Tumor Model

Nude mice were used to study the effect of resveratrol in this study, and they were housed under pathogen-free conditions and given free access to water and food. All experimental protocols were approved by the Ethical Committee of the First Affiliated Hospital of Xi'an Jiaotong University, Xi'an, China. When they were 6–8 weeks of age, 1 × 10^6^ BxPC-3 cells, which were resuspended in a 1:1 (v/v) mixture of culture medium and Matrigel (BD Biosciences, San Jose, CA, USA), were injected into both flanks of nude mice. A subcutaneous tumor model of pancreatic cancer was established. At 1 week after inoculation, the nude mice were randomly divided into the following two groups (five mice per group): blank group (sterile water 100 μl/day, gavage) and resveratrol group (50 mg/kg/day, gavage). At the end of the 5th week of intervention, the nude mice were sacrificed, and the tumor volume was examined. The volume calculation method is (length/2) × (width^2^). H&E staining was used to analyze the tumor samples. A light microscope at ×400 magnification was used to take the representative images of each tumor.

### Statistical Analysis

Each experiment was independently performed at least three times. The data were presented as mean ± SD. Student's *t*-test *via* SPSS (version 15.0; SPSS, Chicago, IL, USA) was used to verify the comparison between two groups. *P*-values < 0.05 were considered as significant.

## Results

### NAF-1 Is Overexpressed in Pancreatic Cancer Cells and Pancreatic Cancer Tissue

To investigate the expression levels of NAF-1, Western blot analysis, real-time PCR, and immunofluorescence were utilized to detect the expression of NAF-1 in several different pancreatic cancer cell lines (Panc-1, MiaPaCa-2, BxPC-3, CF PAC-1, and SW1990) and static pancreatic stellate cell (Q-PSC).

**Figure 1:**
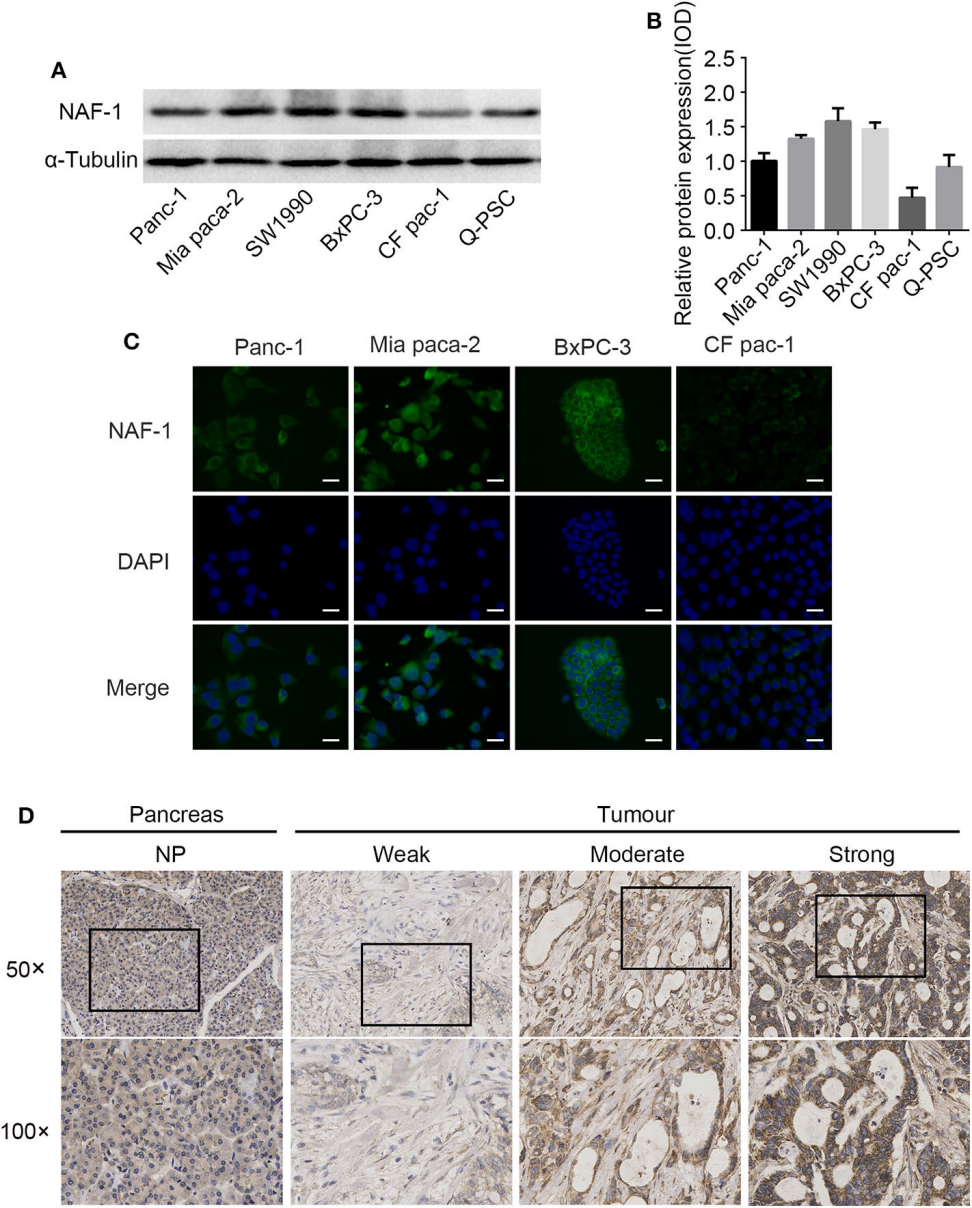
NAF-1 gene expression in pancreatic cancer cells and pancreatic cancer tissue. **(A)** The expression of NAF-1 in different pancreatic cancer cell lines was detected by Western blot. **(B)** The quantified histograms of the Western blots in **(A)**. The bar graph below shows the relative protein expression levels in the cell lines. Column, mean; bar, SD. **(C)** Immunofluorescence staining was performed to estimate the basic expression levels and the locations of NAF-1 in four pancreatic cancer cell lines. NAF-1 staining is shown in green, and nuclear DNA staining by 4′,6-diamidino-2-phenylindole is shown in blue (magnification, ×400; scale bar, 20 μm). **(D)** Immunohistochemistry staining showed the expression pattern of NAF-1 in normal pancreatic tissue and in pancreatic cancer tissue, classified as none, weak, moderate, or strong.

We found that NAF-1 was significantly expressed in the five different pancreatic cancer cell lines tested, but NAF-1 was weakly expressed in CF PAC-1. The other four pancreatic cancer cell lines showed a strong NAF-1 expression. Among the tested cell lines, NAF-1 was also significantly expressed in non-activated Q-PSC, suggesting that NAF-1 also plays an important role in tumor-related PSC, which remains to be further explored and studied (Figures 1A,B). To verify the localization of NAF-1 in cells, immunofluorescence was performed, and the results showed that NAF-1 was mainly located in the cytoplasm of pancreatic cancer cells without nuclear expression (Figure 1C). Based on the experimental results, we selected two pancreatic cancer cells with high NAF-1 expression, namely, Panc-1, and BxPC-3, for our subsequent experiments.

To elucidate the expression pattern of NAF-1 in normal and malignant pancreatic tissues, we analyzed 96 pancreatic specimens, including five normal and 91 pancreatic cancer specimens. In normal pancreatic tissue, there was low coloration in the cytoplasm of pancreatic cells and no coloration in the nucleus, indicating that NAF-1 is expressed at low levels in normal pancreatic tissue. In addition, NAF-1 expression is significantly higher in pancreatic cancer tissue than in normal pancreatic tissue, suggesting that NAF-1 may be related to the progression of pancreatic cancer (Figure 1D).

In addition, in 82/91 cases, immunohistochemical staining confirmed the widespread presence of NAF-1 staining in pancreatic cancer. The expression of NAF-1 in the different pancreatic tissue groups was classified as negative, weak, moderate, and strong. Negative or moderate NAF-1 expression was found in normal pancreatic tissue. Compared with normal pancreatic tissue, the pancreatic cancer tissue had a significantly increased expression of NAF-1 (Figure 1D). It is worth noticing that the expression level of NAF-1 was closely related to the T stage and the TNM stage of pancreatic cancer and was not related to age, gender, tumor differentiation degree, or other factors of the patients (Table 1). These results suggest that NAF-1 may be associated with the progression of pancreatic cancer and is a valuable biomarker.

### Inhibition of NAF-1 Significantly Reduces the Invasion of Pancreatic Cancer Cells

To study the effect of NAF-1 on the invasion and the migration of pancreatic cancer cells *in vitro*, we first inhibited the expression of NAF-1 by shRNA in two pancreatic cancer cell lines (Panc-1 and BxPC-3). Then, a cell scratch assay and a transwell assay were used.

**Figure 2:**
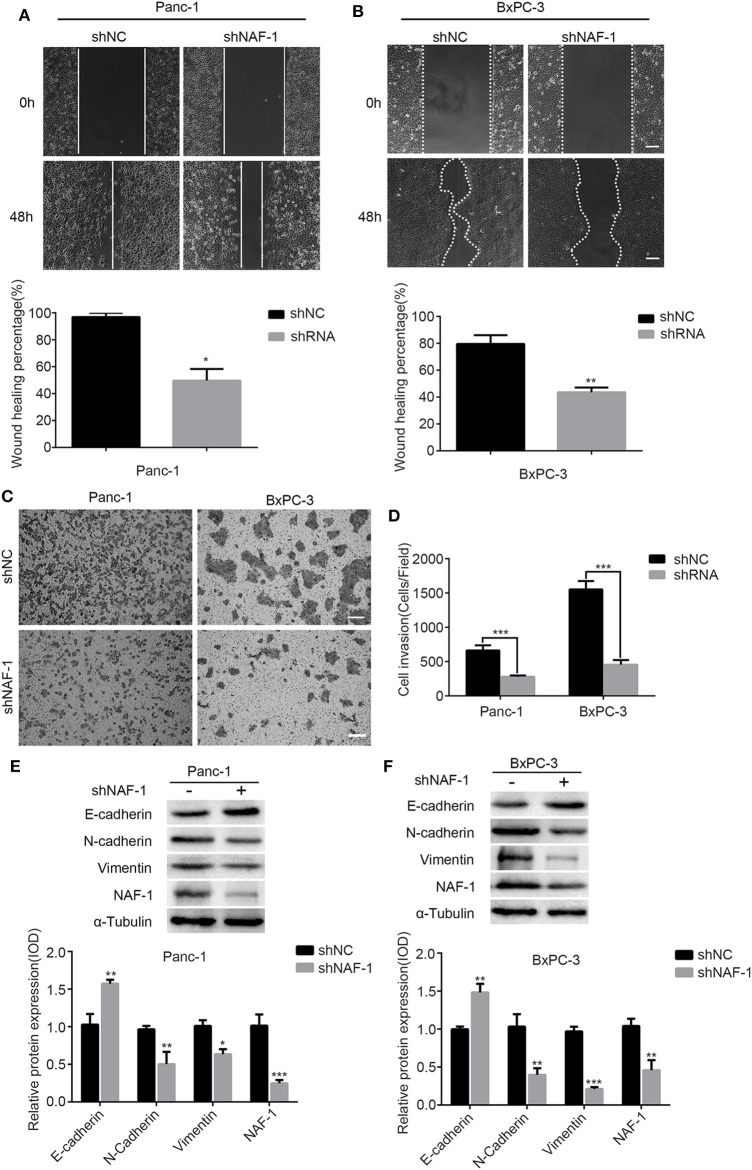
The inhibition of NAF-1 significantly reduced the invasion and the migration of pancreatic cancer cells. **(A,B)** A scratch assay was used to detect the effect of shNAF-1 intervention on the migration ability of Panc-1 and BxPC-3 cells; statistical analyses of the percent cell migration distance are shown (**p < 0.01). Scale bar = 100 μm. **(C,D)** Migration of two pancreatic cancer cell lines in the shNAF-1-positive knockdown group and the shNC control group after 24 h in a Transwell chamber precoated with matrix gel. Crystal violet staining showed that the cells in the two groups migrated to the subcompartment membrane within 24 h; the statistical analysis of the assay is shown. Scale bar = 100 μm. **(E,F)** Panc-1 and BxPC-3 cells were cultured under the same normal conditions for 48 h. Western blot was used to detect the changes in epithelial–mesenchymal transition-related indicators (*P < 0.05, **p < 0.01, ***p < 0.001).

Cell migration ability was first tested by the scratch assay. In this experiment, the Panc-1 and the BxPC-3 cells were each divided into two groups, the shNC and the shNAF-1 interference groups. The cells in the negative control group were transfected with shNC, the scrambled shRNA, and the cells in the control group (NC group) were transfected with shNAF-1, which had been verified for knockdown efficiency. The cells of both groups were cultured without serum for 48 h after the scratch treatment. The area of the scratch edge between two blanks was recorded at 0, 24, and 48 h for each group. The cell migration rate was calculated as the percentage of the difference in the initial (0 h) area and the areas at different time points. Statistical analysis was conducted on the results of three experiments, and a histogram was drawn. The results showed that the degree of cell migration from the scratch edge to the center was lower in the NAF-1 knockdown group than in the control group. The cell migration rate of the two groups was significantly different (*p* < 0.01) (Figures 2A,B).

To further confirm the effect of NAF-1 on the invasion ability of pancreatic cancer cells, we used a cell invasion assay to detect the invasion ability of the pancreatic cancer cell lines Panc-1 and BxPC-3. It shows that the number of cells in the experimental group was significantly less than that in the control group, and the ratios of cells in the two groups were significantly different (*p* < 0.01) (Figures 2C,D). It is known that the occurrence of EMT is an important mechanism, leading to the invasion and the metastasis of tumor cells. Therefore, the markers related to EMT (E-cadherin, N-cadherin, and Vimentin) were examined by Western blot analysis. Image J software was used to quantify the expression of NAF-1 and EMT-related proteins. We found that the epithelial markers, such as E-cadherin, were increased, while the interstitial markers, such as N-cadherin and Vimentin, were significantly decreased in the shNAF-1 knockdown group compared with those in the control group (Figures 2E,F). These results suggest that the inhibition of NAF-1 significantly reduces the invasion of pancreatic cancer cells.

### Inhibition of NAF-1 Significantly Inhibits Stem Cell Characteristics

For many years, the post-operative recurrence and metastasis of pancreatic cancer had been a major problem in the treatment of pancreatic cancer. Most researchers attribute these processes to the existence of PCSCs. A previous study found that the stem cells in pancreatic cancer have a complex association with chemotherapy resistance (14). Therefore, identifying whether NAF-1 is a key signaling molecule that affects the characteristics of PCSCs and developing interventions against this key molecule may be of great significance for inhibiting the progression of pancreatic cancer and preventing resistance to chemotherapy.

**Figure 3:**
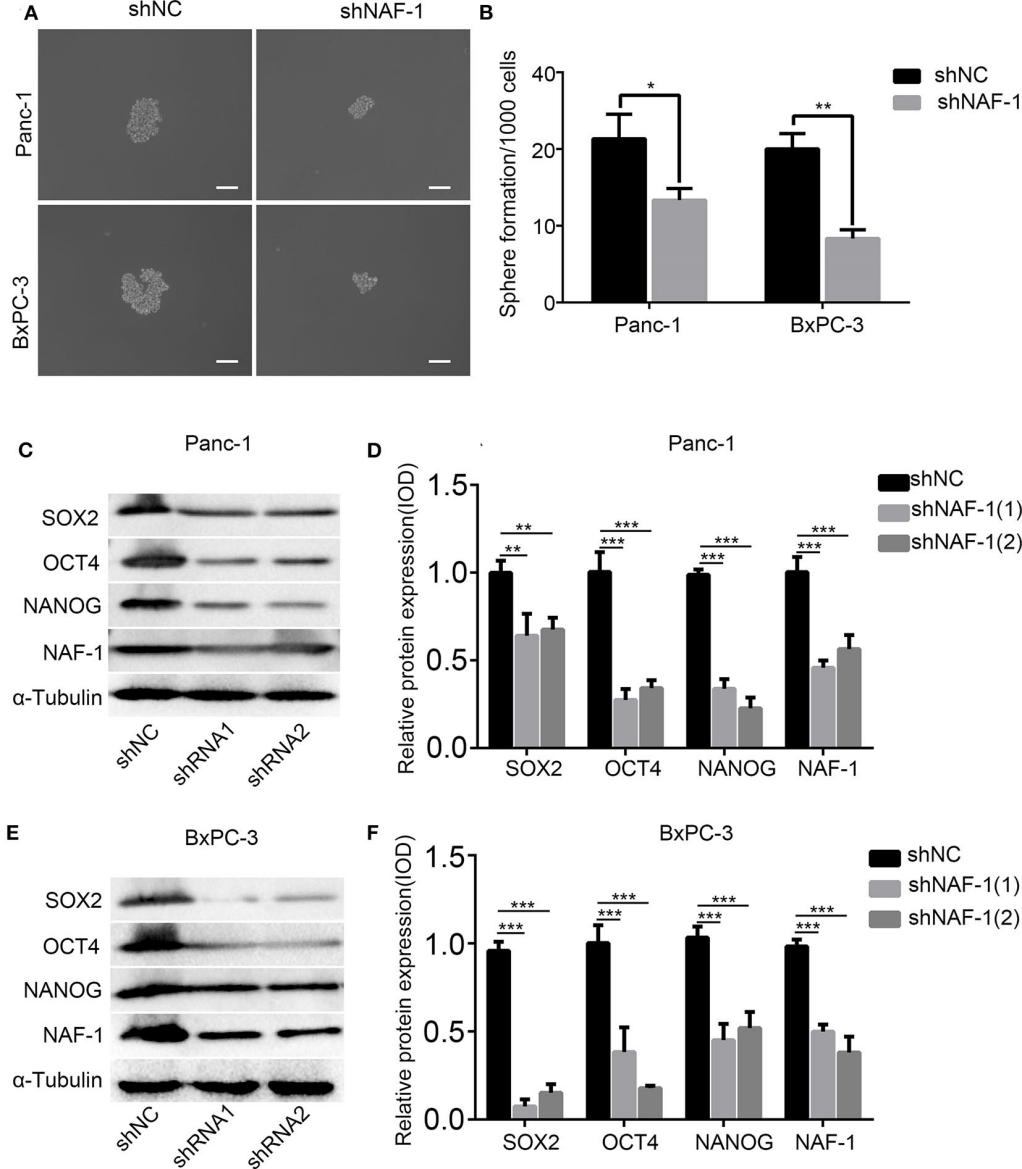
Inhibition of NAF-1 significantly inhibited the stem cell characteristics. **(A)** The pancreatic cancer cells treated with shNC or shNAF-1 were inoculated in serum-free Dulbecco's Modified Eagle's Medium/F12 medium (containing 20 ng/ml epidermal growth factor, 20 ng/ml fibroblast growth factor, and 1% B27) for 1 week to determine the ability of the cancer cells to form spheroids. The representative bright-field microscopy images of tumor spheres after shNAF-1 intervention in Panc-1 and BxPC-3 are shown. Scale bar = 100 μm. **(B)** Quantification of the number of spheres in Panc-1 and BxPC-3 treated with shNC or shNAF-1. **(C,E)** Western blot showed the effect of shNAF-1 on the protein expression levels of stem cell indexes in Panc-1 and BxPC-3 after intervention. **(D,F)** Statistical analyses of **(C,E)** (column, mean; bar, SD; *p < 0.05, **p < 0.01, ***p < 0.001).

Through a spheroid formation assay, we found that the spheroid of pancreatic cancer cells was significantly decreased after NAF-1 inhibition. The number of stem cell spheroids in the control group was significantly increased compared to that in the experimental group (Figures 3A,B). SOX2, NANOG, and OCT4 are markers of stem cells. After treating with shRNA, Western blot analysis was used to detect the expression levels of stem cell markers in Panc-1 (Figures 3C,D) and BxPC-3 (Figures 3E,F). The expression levels of all the three markers were reduced after the NAF-1 knockdown, which was consistent with the results of the spheroid formation assay. Taken together, our results showed that the downregulation of NAF-1 expression can reduce the stem cell characteristics of pancreatic cancer cells.

### Resveratrol Inhibits the Invasion of Pancreatic Cancer Cells

The remarkable feature of malignant tumor cells is the acquisition of strong invasion and metastasis abilities. Moreover, these invasion and metastasis tendencies are also one of the important reasons for the poor prognosis of pancreatic cancer (15). As a polyphenol plant anti-toxin, resveratrol has many biological effects, including anti-tumor effects. Additionally, whether resveratrol has a similar effect in pancreatic cancer is not entirely clear. To investigate this effect of resveratrol, we treated Panc-1 and BxPC-3 cells with different concentrations of resveratrol and used an invasion assay and a scratch assay to detect the invasion and migration ability of pancreatic cancer cells.

**Figure 4:**
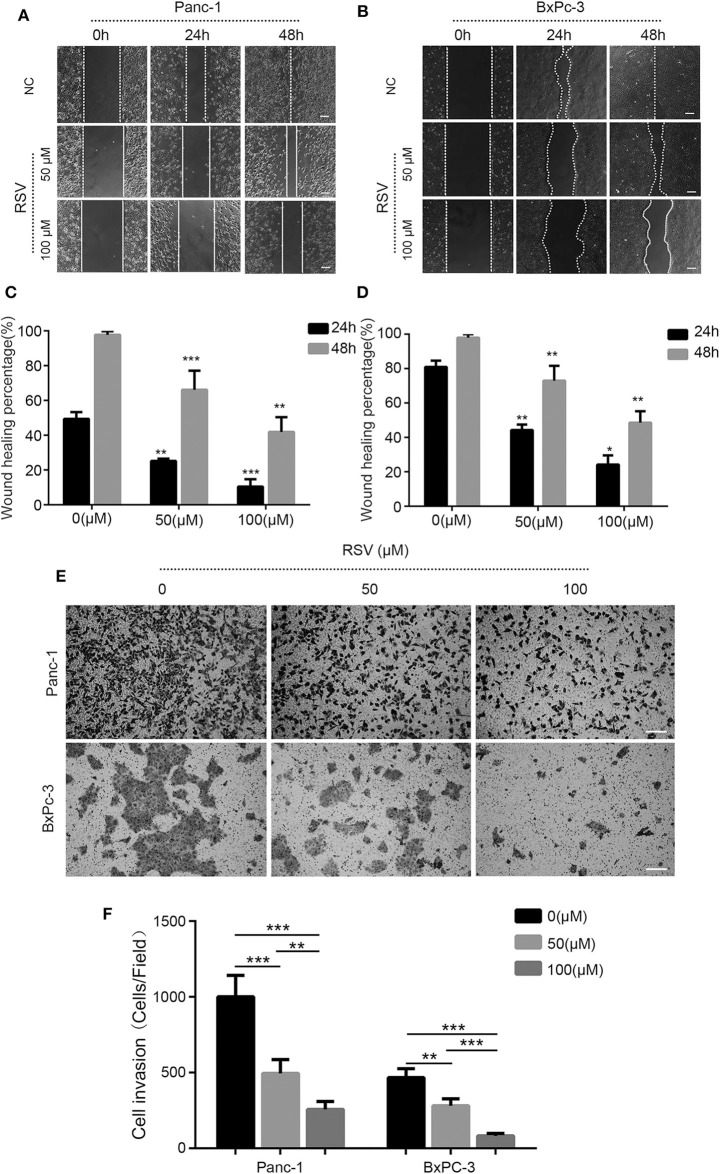
Resveratrol inhibits the invasion and the migration of pancreatic cancer cells. **(A–D)** A scratch assay was used to detect the effect of resveratrol intervention on the migration ability of Panc-1 and BxPC-3 cells; statistical analyses of the percent cell migration distance are shown (*p < 0.05, **p < 0.01, ***p < 0.001). Scale bar = 100 μm. **(E,F)** Migration of two pancreatic cancer cells in the resveratrol-treated knockdown group and the shNC control group after 24 h in a Transwell chamber precoated with matrix gel. Crystal violet staining showed that the cells in the two groups migrated to the subcompartment membrane within 24 h; the statistical analysis of the assay is shown. Column, mean; bar, SD (*p < 0.05, **p < 0.01, ***p < 0.001). Scale bar = 100 μm.

Based on previous experiments (16), the scratch assay showed that both 50 and 100 μM of resveratrol could inhibit the migration ability of pancreatic cancer cells (Figures 4A,B); the cell migration ability of the experimental group was significantly decreased compared with that of the negative control group at the same time point (Figures 4C,D). In addition, we used 50 and 100 μM resveratrol to treat Panc-1 and BxPC-3 cells for 24 and 48 h, respectively. We found that the number of cells passing through the membrane was decreased with resveratrol treatment, indicating that resveratrol can significantly inhibit the invasion ability of pancreatic cancer cells (Figures 4E,F). Taken together, our results indicate that resveratrol inhibits the invasion of pancreatic cancer cells.

### Resveratrol Inhibits the Stem Cell Characteristics of Pancreatic Cancer

**Figure 5:**
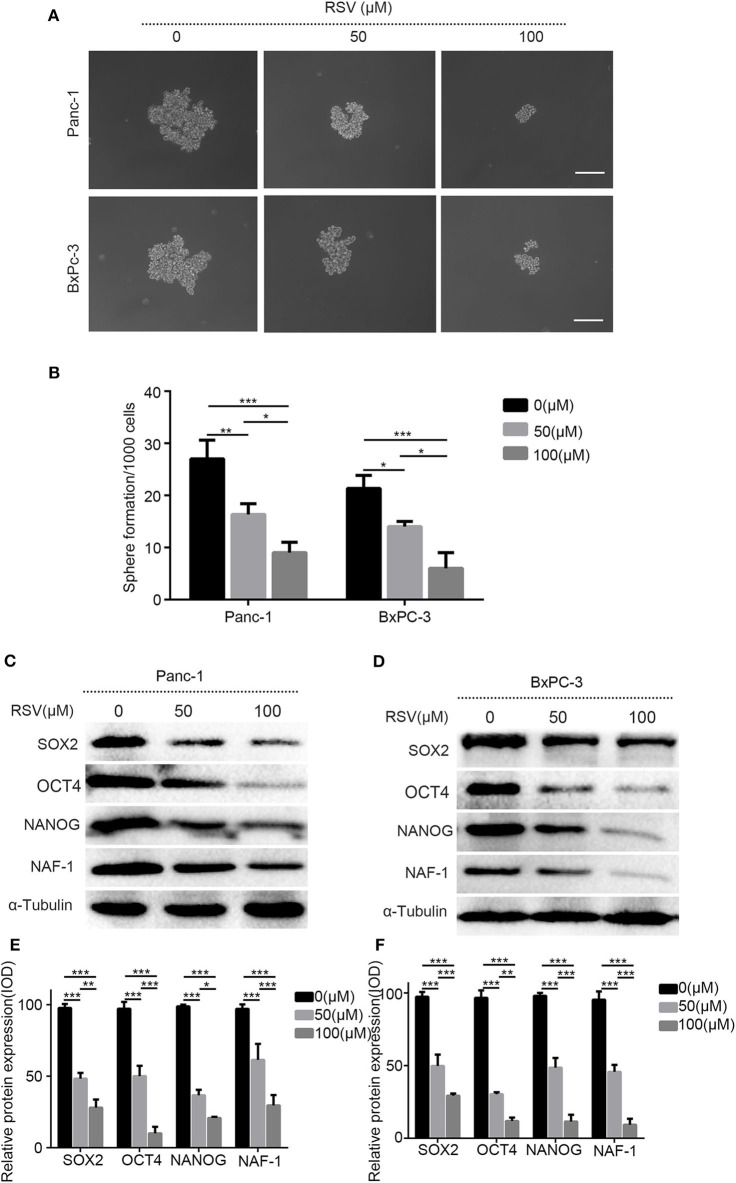
The effects of resveratrol on the characteristics of pancreatic cancer stem cells. **(A)** The pancreatic cancer cells treated with resveratrol and the representative bright-field microscopy images of tumor spheres after resveratrol intervention in Panc-1 and BxPC-3 are shown. Scale bar = 100 μm. **(B)** Quantification of the number of spheres in Panc-1 and BxPC-3 treated with resveratrol. **(C,D)** Western blot showed the effect of resveratrol on the protein expression levels of cell stem indexes in Panc-1 and BxPC-3 after the intervention. **(E,F)** Statistical plots of **(C,D)** (column, mean; bar, SD; *p < 0.05, **p < 0.01, ***p < 0.001).

To study the effects of resveratrol on the characteristics of PCSCs, we conducted a spheroid formation assay. Panc-1 and BxPC-3 were pretreated with resveratrol at different concentrations (50 and 100 μM) for 24 h and then inoculated in serum-free DMEM/F12 medium (containing 20 ng/ml EGF, 20 ng/ml FGF, and 1% B27) for 1 week to determine the ability of the cancer cells to form pellets. The results showed that resveratrol, at different concentrations, could inhibit the ability of the pancreatic cancer cells to form spheroids, and the number of stem spheres in the experimental group was significantly lower than that in the control group (Figures 5A,B). In addition, Western blot analysis was used to detect the expression levels of the stem cell markers SOX2, NANOG, and OCT4 after treating with resveratrol at different concentrations (50 and 100 μM) for 24 h (Figures 5C,D). The expression levels of the three markers were reduced after the resveratrol intervention (Figures 5E,F), and the results were consistent with the results of the spheroid formation assay. In summary, our results reveal that resveratrol can inhibit the stem cell characteristics of pancreatic cancer cells.

### Resveratrol Inhibits the Stem Cell Characteristics and the Migration of Pancreatic Cancer *via* Suppressing NAF-1

**Figure 6:**
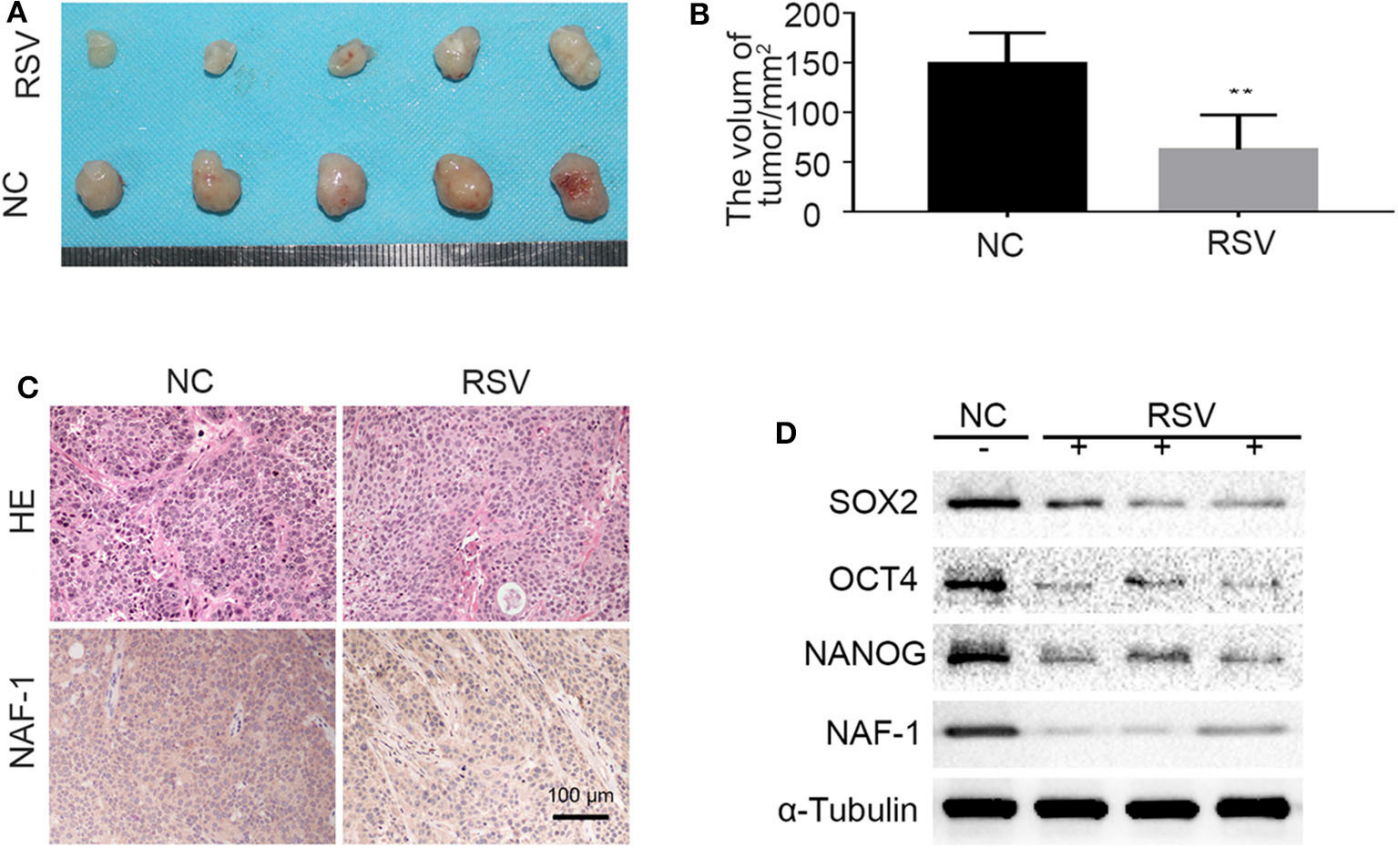
The effects of resveratrol on the stem cell characteristics of pancreatic cancer. **(A,B)** The effect of resveratrol on the growth of pancreatic cancer tumors subcutaneously transplanted in nude mice. [The mice in the control and resveratrol groups were treated daily with vehicle or resveratrol (50 mg/kg) by gavage, respectively] (**P < 0.01). **(C)** Immunohistochemical staining shows the expression level of NAF-1 in the different groups. **(D)** Proteins were extracted from the tissue, and the expression of NAF-1 and stem cell-related markers in the tumor tissue of each group was verified by Western blot.

To confirm the results *in vitro*, we constructed a subcutaneous tumor model of pancreatic cancer in nude mice by inoculating pancreatic cancer cells (1 × 10^6^) under the skin of the back. After 1 week of inoculation, the nude mice were randomly divided into two groups, the blank group and the resveratrol group. At the end of the 5th week of intervention, the nude mice were sacrificed by the vertebral detachment method, and the tumor volume was examined and recorded. The results indicate that the growth of pancreatic cancer tumors can be inhibited by resveratrol (Figures 6A,B).

In addition, H&E and immunohistochemical staining were performed on pancreatic cancer tumor specimens subcutaneously transplanted in nude mice. The results showed that the tumor tissue of the control group (NC group) contained many interstitial components and the cancer nests in the NC group were distributed in a focus-like pattern surrounded by interstitium. In the resveratrol group, the tumor tissues were similar to those in the control group and contained many interstitial components. However, the NAF-1 staining of the resveratrol group was significantly weaker than that of the control group, indicating that the expression of NAF-1 was inhibited under resveratrol intervention (Figure 6C). Furthermore, we extracted proteins from the tumor samples of the control group and the resveratrol group, and Western blot was performed to detect changes in NAF-1 and stem cell-related markers. The results showed that, compared with the control group, the resveratrol group had significantly decreased expression levels of NAF-1 and stem cell-related markers (Figure 6D). These results suggest that resveratrol can inhibit the stem cell characteristics of pancreatic cancer and further inhibit the progression of pancreatic cancer.

**Figure 7:**
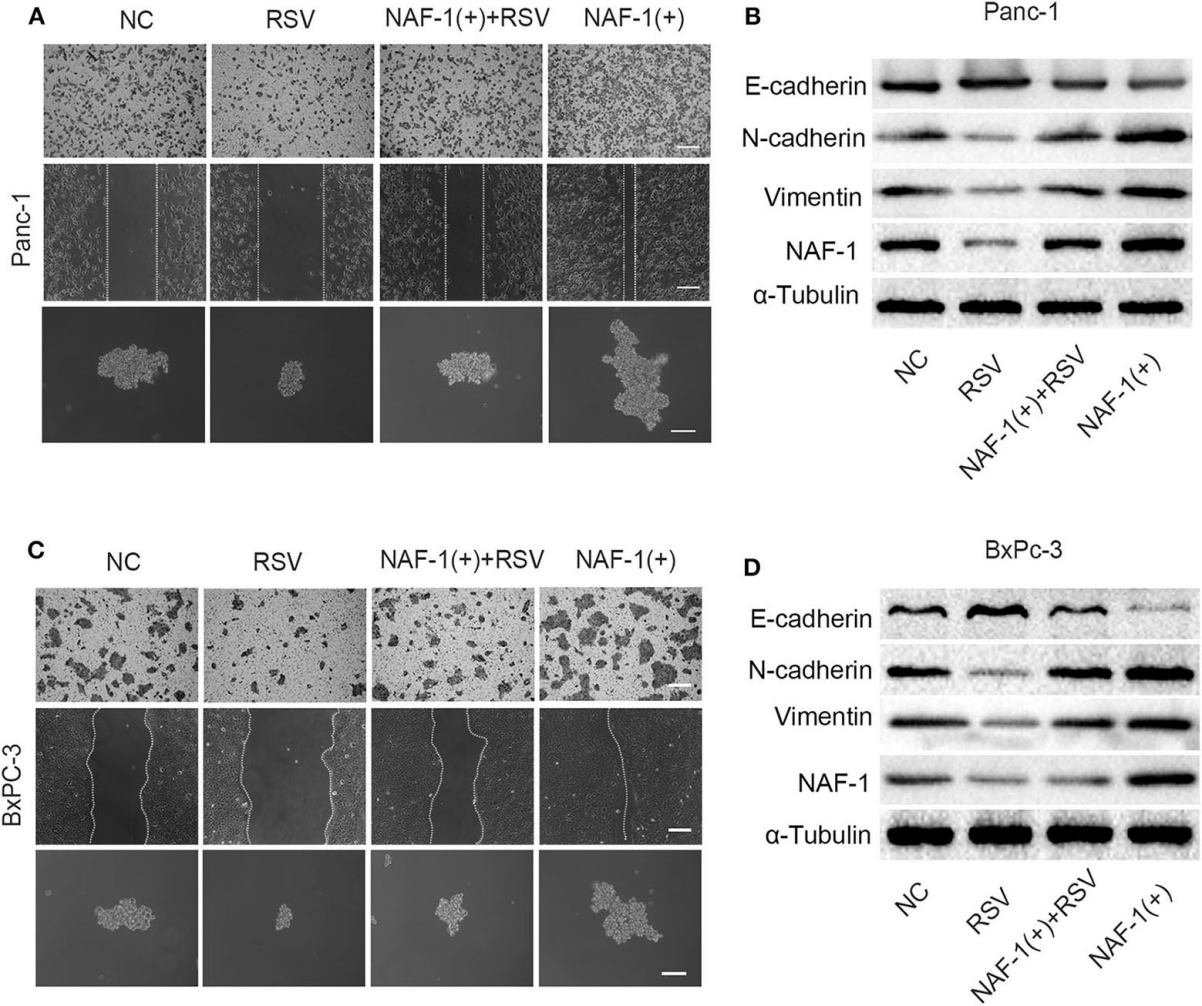
**(A)** Effects of resveratrol combined with NAF-1(+) on the invasion, metastasis, and stem cell characteristics of Panc-1 cell line. Scale bar =100 μm. **(B)** The pancreatic cancer cells (Panc-1 cells) were treated with resveratrol alone or resveratrol combined with NAF-1(+), and Western blot was used to detect the expression of epithelial–mesenchymal transition (EMT)-related indicators. **(C)** Effects of resveratrol combined with NAF-1(+) on the invasion, metastasis, and stem cell characteristics of BxPC-3 cell line. Scale bar =100 μm. **(D)** Pancreatic cancer cells (BxPC-3 cells) were treated with resveratrol alone or resveratrol combined with NAF-1(+), and Western blot was used to detect the expression of EMT-related indicators.

We constructed a pancreatic cancer cell line with increased NAF-1 expression using lentivirus. The experiment was divided into the following four groups: control group, resveratrol group, resveratrol combined with NAF-1(+) group, and NAF-1(+) group. In contrast, under the resveratrol intervention, the above effects were inhibited. Interestingly, we found that resveratrol supplementation can reverse the characteristics of NAF-1 expression in pancreatic cancer (Figures 7A,C). Western blot was used to detect changes in EMT-related indicators in the groups, and the results were consistent with the above results (Figures 7B,D). Therefore, we conclude that resveratrol inhibited the invasion of pancreatic cancer and stem cell characteristics by inhibiting NAF-1.

## Discussion

Pancreatic cancer is a highly malignant disease, with a 5-year survival rate of < 9% (1). Despite improvements in treatment, including surgical techniques and local and systemic adjuvant therapy, the mortality rate of pancreatic cancer remains high (17). Pancreatic cancer is one of the leading causes of cancer-related death in developed countries.

In the past decade, considerable progress has been made in understanding the changes in genes involved in local and systemic tumor growth, with the most important changes occurring in genes that regulate cell cycle progression, extracellular matrix homeostasis, and cell migration. However, the molecular mechanism of pancreatic cancer remains unclear. It is generally accepted that invasion and tumor metastasis are closely related (18). In recent years, NAF-1 has gained increasing interest, and it was found that NAF-1 expression is increased in a variety of tumors, including gastric cancer (19), prostate cancer (20), cervical cancer (21), liver cancer (22), and laryngeal cancer. Elevated NAF-1 expression often indicates a high recurrence rate and poor prognosis. In this study, we found that NAF-1 is positively expressed in pancreatic cancer tissue. A histopathological analysis of clinical specimens revealed that the NAF-1 expression levels are significantly increased in pancreatic cancer tissue compared with those in normal pancreatic tissue. According to the statistical analysis of the clinicopathological results, the expression level of NAF-1 was related to the T stage of pancreatic cancer, but not to age, gender, tumor differentiation degree, or other factors. These results suggest that NAF-1 may accelerate the progression of pancreatic cancer and is a valuable biomarker (23). We also found that it seemed that the expression of NAF-1 was not always higher in pancreatic cancer cells. The reason may be that the pancreatic cancer cell lines originated from different patients and different parts of these patients. Each cell line has a unique genetic background. The biologic and pathologic characteristics of the tumor cell line resulted in different abilities of proliferation, invasion, and other malignant phenotypes. Besides that, these characteristics also resulted in different gene expression levels in different cell lines (24).

From a clinical perspective, CSCs are important because they are resistant to a variety of chemotherapies and radiation (7). CSCs were first identified in hematological cancers, followed by their detection in practically all solid tumors, including pancreatic ductal adenocarcinoma (25). In this context, it is very important to better understand the characteristics of pancreatic CSCs in order to develop new therapies that target these cells. Our study showed that the inhibition of NAF-1 can effectively inhibit the stem cell characteristics of pancreatic cancer and the invasion and the metastasis of pancreatic cancer, thereby inhibiting the progression of pancreatic cancer. It is well-known that NAF-1 mainly exists in the endoplasmic reticulum and mitochondrial outer membrane and is necessary for maintaining mitochondrial integrity (26). When the NAF-1 expression is defective, mitochondrial degeneration and functional decline occur, and then mitochondrial-related cell death is induced (27, 28). In addition, NAF-1 is involved in regulating Becnl-1-mediated autophagy (29). CSCs express a high level of reactive oxygen species (ROS) scavenger proteins, which is related to the low level of intracellular ROS (30). There is a large difference in the energy and the metabolism of CSCs and ultimately differentiated stem cells. In addition, the stem cells maintain their pluripotency by relying on At93 autophagy to participate in mitochondrial homeostasis regulation (31). Therefore, NAF-1 is likely to be an important part of the metabolic regulation of tumor stem cells.

Several mechanisms have been reported to be involved in the regulation of pancreatic cancer invasion and metastasis, including EMT (32, 33). EMT is a process in which cancer cells acquire mesenchymal features and lose epithelial phenotypes at the same time. This process is accompanied by a decreased expression of E-cadherin and an increased expression of N-cadherin and vimentin. Tumor cells with a mesenchymal phenotype show marked plasticity, which contributes to cell deformation and promotes cell migration and metastasis. The EMT and the invasion behavior of pancreatic cancer cells are regulated by some transcription factors, such as Snail, Slug and Twist. In this study, we found that the inhibition of NAF-1 can inhibit the EMT characteristics. These results suggest that NAF-1 plays an important role in the development of pancreatic cancer. Therefore, a targeted molecular intervention against NAF-1 may provide a positive theoretical basis for the development of a novel treatment for pancreatic cancer in the future. The EMT process endows cancer cells with stem cell-like characteristics to achieve self-renewal and enhance their ability of proliferation and metastases (34). Therefore, there is a direct relationship between the EMT process and CSCs. The results in our study showed that the inhibition of NAF-1 can effectively inhibit the EMT and the stem cell characteristics of pancreatic cancer cells, suggesting that NAF-1 intervention may be an effective compound for inhibiting pancreatic cancer metastasis by targeting PCSCs and EMT. Although the molecular markers that can be used to label tumor stem cell-like phenotypes were not very clear yet, SOX2, OCT4, and NANOG, which are transcription factors, have been confirmed to be the main regulatory factors to maintain tumor stem cell-like phenotypes (35). SOX2, OCT4, and NANOG are overexpressed in poorly differentiated malignant tumors and their expression overlaps with the characteristics of embryonic stem cells. This study confirmed the discovery that NAF-1 signaling plays an important role in the regulation of pancreatic cancer EMT and stem cells.

Resveratrol has a variety of biological effects, such as anti-oxidant, anti-inflammatory, cardiac and neuro protection, and anti-diabetic effects (3). Besides that, resveratrol plays an important role in stem cell-related mechanisms (36). Resveratrol can modulate non-cancer cells in the tumor microenvironment and inhibit the cellular reprogramming of non-cancer cells to inhibit metastasis and suppress resistance to anti-cancer therapy in cancer cells (37). In recent years, many studies have shown that resveratrol can directly inhibit the proliferation of tumor cells, induce growth inhibition, cell cycle arrest, and apoptosis, and reduce tumor cell viability (4). However, the specific molecular mechanism of resveratrol in cancer treatment is still unclear, and further research is needed. Resveratrol can significantly inhibit various tumor cells, such as hepatocellular carcinoma, leukemia, and breast cancer, through various mechanisms (3, 38). In pancreatic cancer models, resveratrol has been considered to be a protective or therapeutic drug and has been shown to have synergistic anti-tumor effects, in combination with gemcitabine (16). In this study, we also found that resveratrol significantly inhibits stem cell characteristics as well as the invasion and the migration of pancreatic cancer cells by inhibiting NAF-1. It is well-known that NAF-1 is an important regulator of intracellular oxidative stress and autophagy (39), and resveratrol can induce apoptosis by regulating intracellular autophagy (40). Therefore, whether autophagy plays a role in mediating the transmission of information between resveratrol and NAF-1 remains to be further explored. Furthermore, studies have shown that an increased ROS level of malignant tumor cells plays an important role in the occurrence and the development of cancer by promoting cell proliferation, survival, invasion, and metastasis (41). Resveratrol regulates the activity of intracellular anti-oxidant enzymes through ROS, which may contribute to its anti-cancer effect. The main molecular mechanisms of resveratrol as a tumor suppressor include the following: (1) regulating the activation of mitochondria and the caspase cascade enzyme system, (2) upregulating cyclin-dependent kinase inhibitors, tumor suppressor genes, death-inducing cytokines, and their receptors, (3) downregulating the expression of surviving, cFLIP, cIAPs, and anti-apoptotic proteins (bcl-2 and bcl-xl) related to the occurrence of chemotherapy resistance (42), (4) activating adenosine 5′-monophosphate-activated protein kinase, (5) inhibiting MAPK, PI3K/Akt (43), Sonic hedgehog (44), Hippo-YAP (16), PKC, EGFR kinase, and NF-kappaB, and (6) activating protein-1 (45), HIF-1 (46), and STAT3 (47), *etc*. NAF-1 may play an important role in resveratrol-mediated mitochondrial metabolism; however, the exact molecular mechanisms need to be further studied.

Our present study demonstrates that the inhibition of NAF-1 significantly inhibits the stem cell characteristics as well as the invasion and migration abilities of pancreatic cancer cells. Furthermore, resveratrol, as a natural anti-tumor drug, can inhibit the progression and the stem cell characteristics of pancreatic cancer *via* NAF-1. These findings elucidate the important role of NAF-1 in the resveratrol-mediated modulation of tumor progression and may hold promise as a potential therapeutic target against pancreatic cancer.

## Data Availability Statement

All datasets generated for this study are included in the article/Supplementary Material.

## Ethics Statement

The studies involving human participants were reviewed and approved by the Ethical Committee of the First Affiliated Hospital, Xi'an Jiaotong University, Xi'an, China. The patients/participants provided their written informed consent to participate in this study. The animal study was reviewed and approved by the Ethical Committee of the First Affiliated Hospital, Xi'an Jiaotong University, Xi'an, China.

## Author Contributions

LC, JM, QM, and EW contributed to the conception and design of the study. LC, TQ, YX, and WQ contributed to the development of the methodology. LC, TQ, YX, WQ, and JL contributed to the acquisition and analysis of data. LC, TQ, JM, QM, ZWu, ZWa, and LW contributed to the writing, review, and/or revision of the manuscript. LC, TQ, ZWu, JM, QM, and LW provided administrative, technical, or material support. LC, JM, QM, QX, and WD supervised the study. LC and TQ have contributed equally to this work. All authors contributed to the article and approved the submitted version.

### Conflict of Interest

The authors declare that the research was conducted in the absence of any commercial or financial relationships that could be construed as a potential conflict of interest.
