# Supplementary material for: NAF-1 Inhibition by Resveratrol Suppresses Cancer Stem Cell-Like Properties and the Invasion of Pancreatic Cancer
Source: Front Oncol. 2020 Jul 16;10:1038. doi: 10.3389/fonc.2020.01038 (PMC7378530; doi:10.3389/fonc.2020.01038)
Supplement: Supplementary file 1 [file Data_Sheet_1.docx]

*This digit record is for Figure 1A*

*
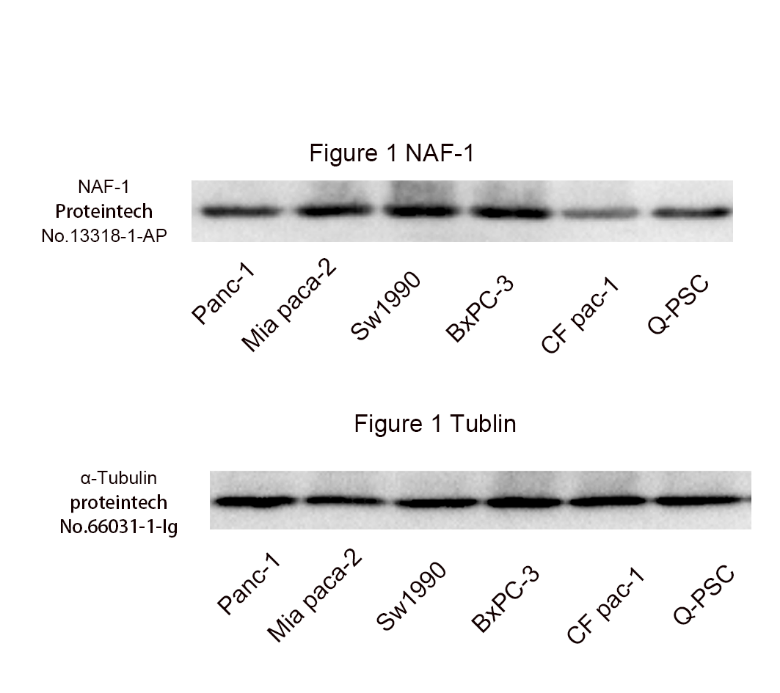
*

*This digit record is for Figure 2*

*
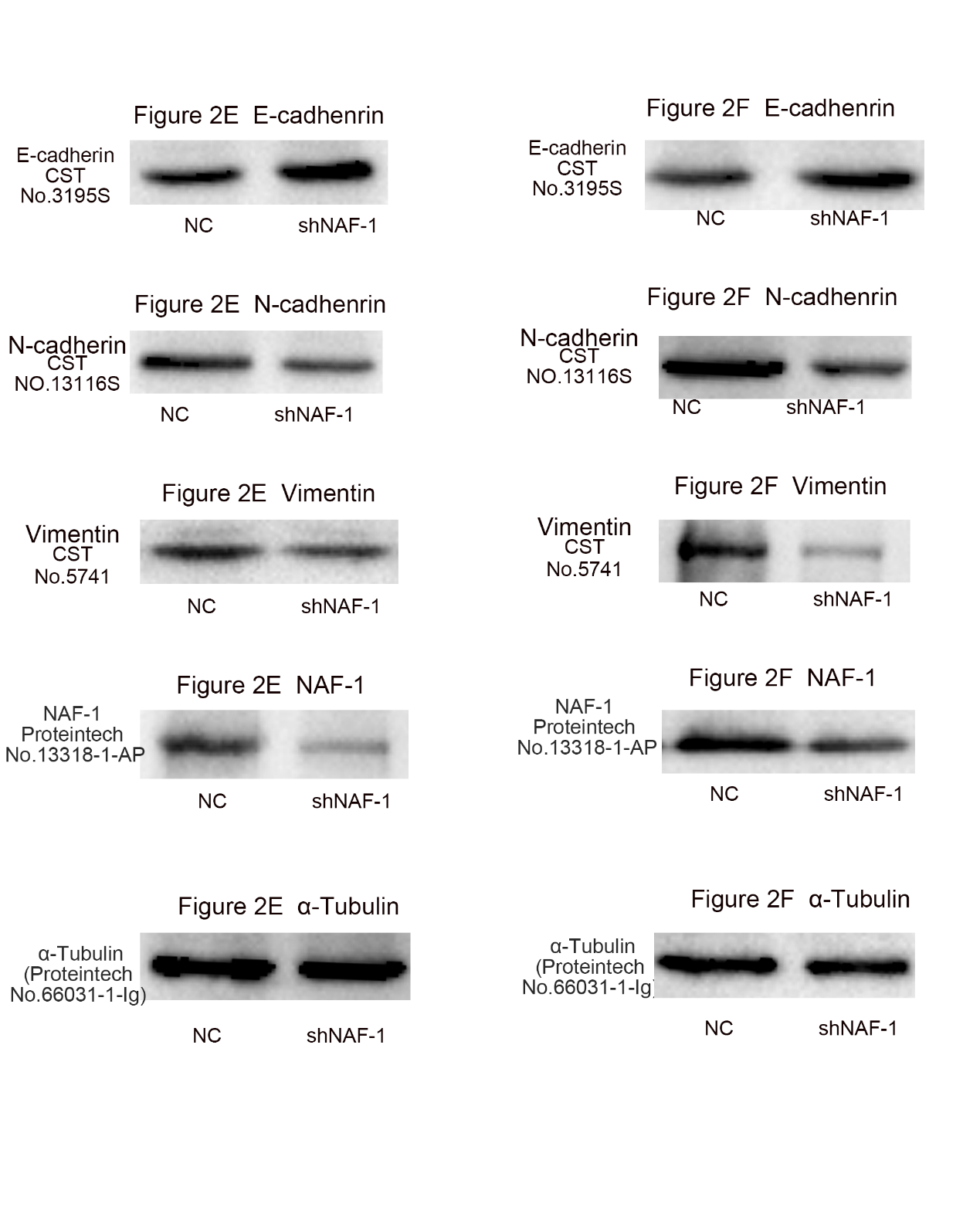
*

*This digit record is for Figure 3C and 3E.*


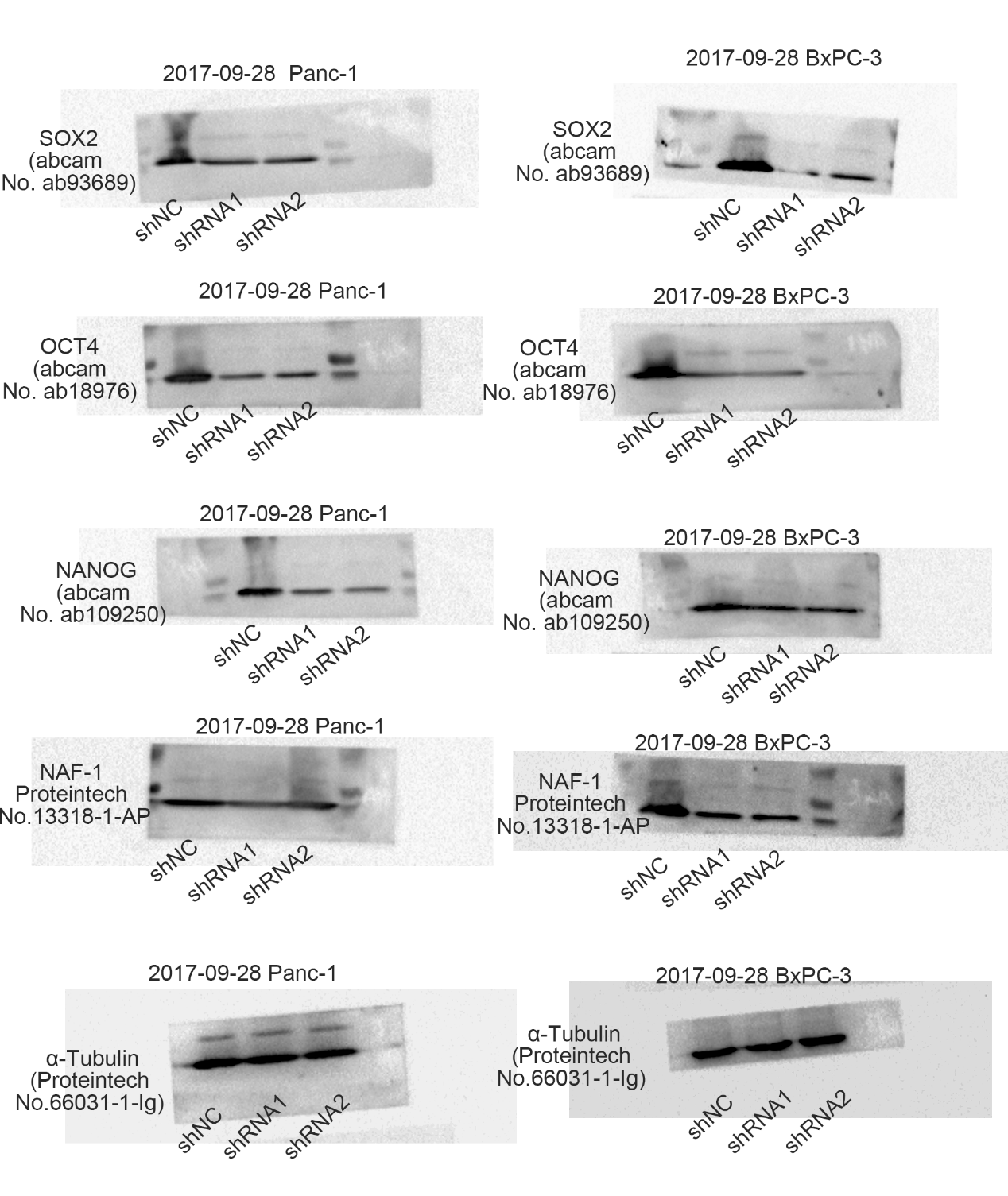


*This digit record is for Figure 5*


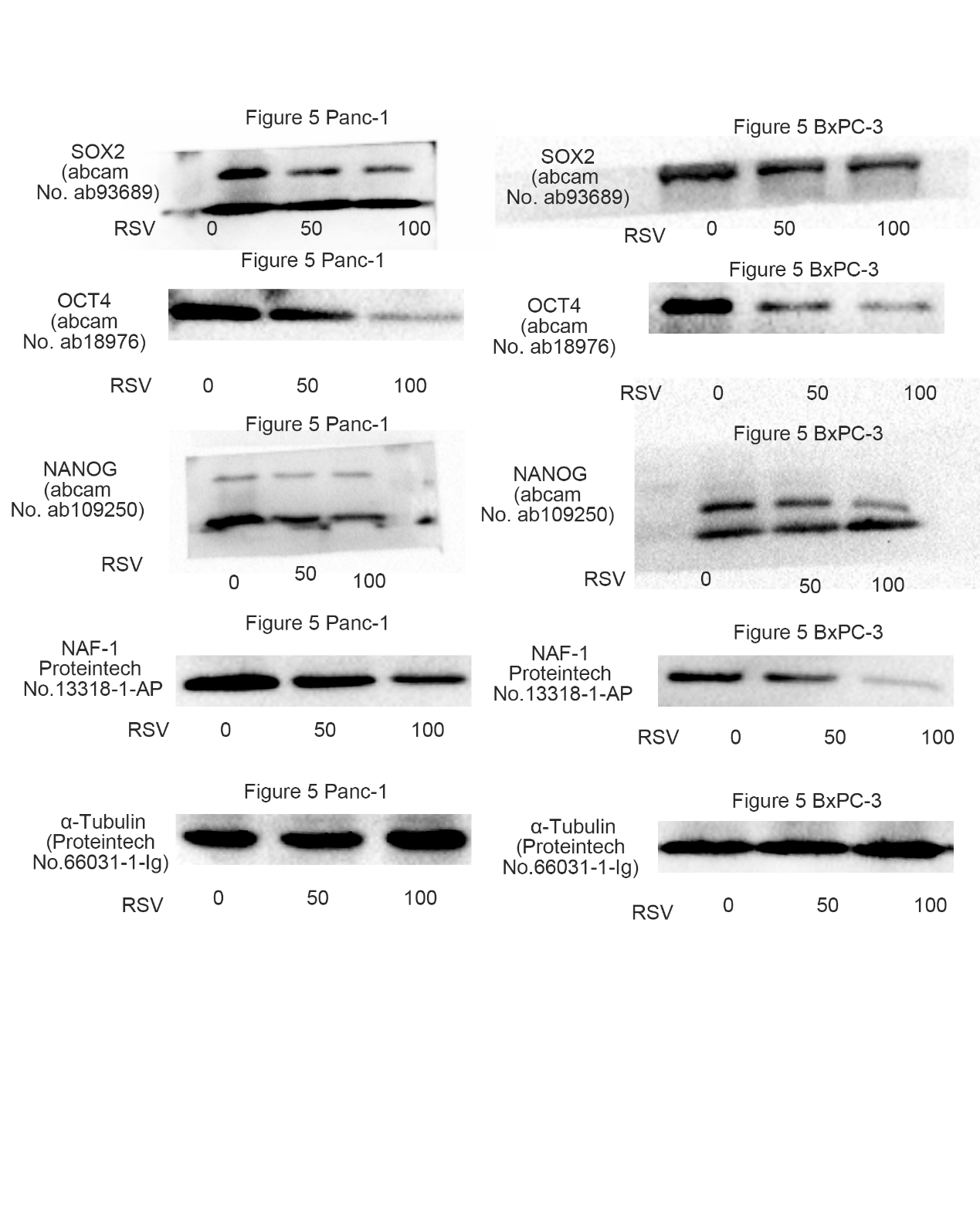


*This digit record is for Figure 6*

*
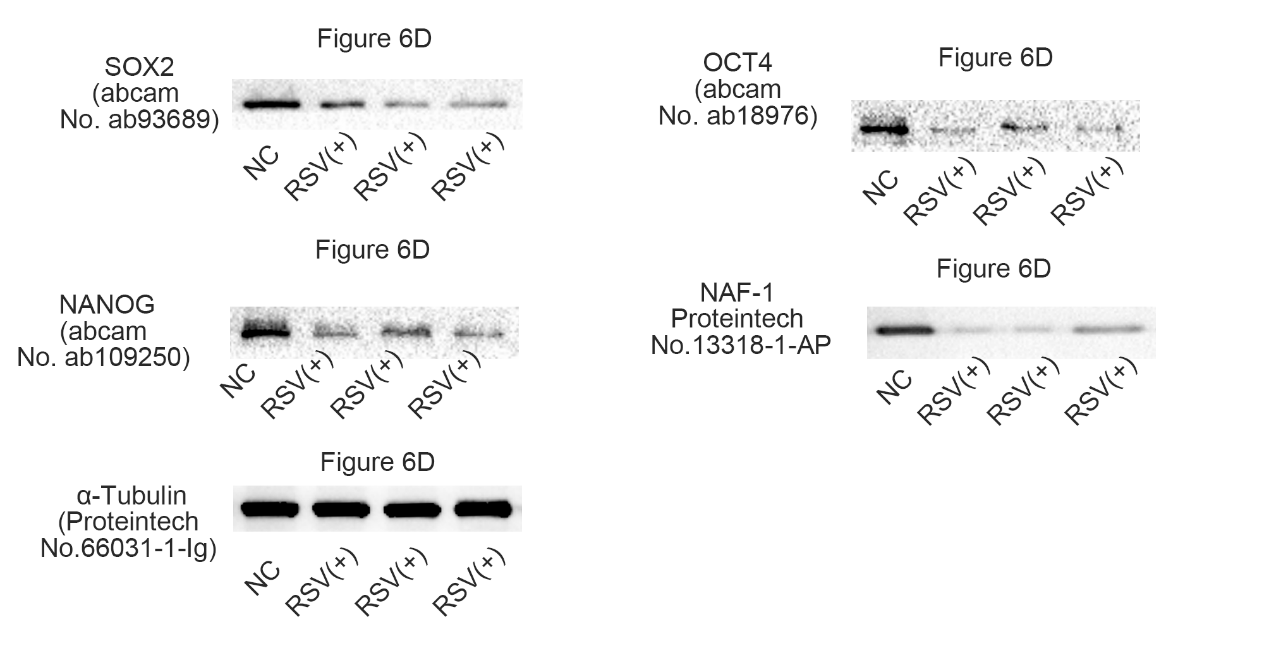
*

*This digit record is for Figure 7*


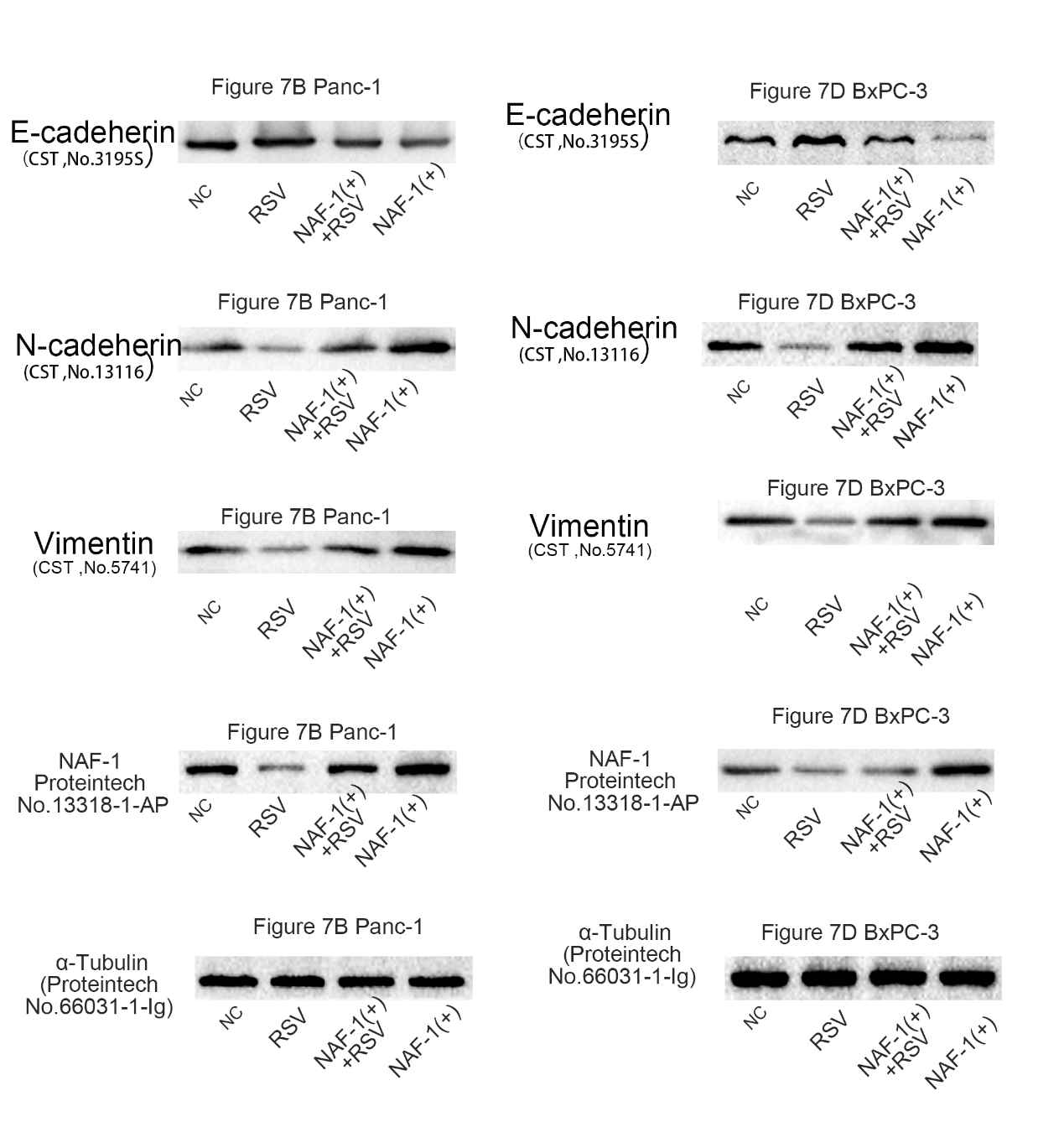

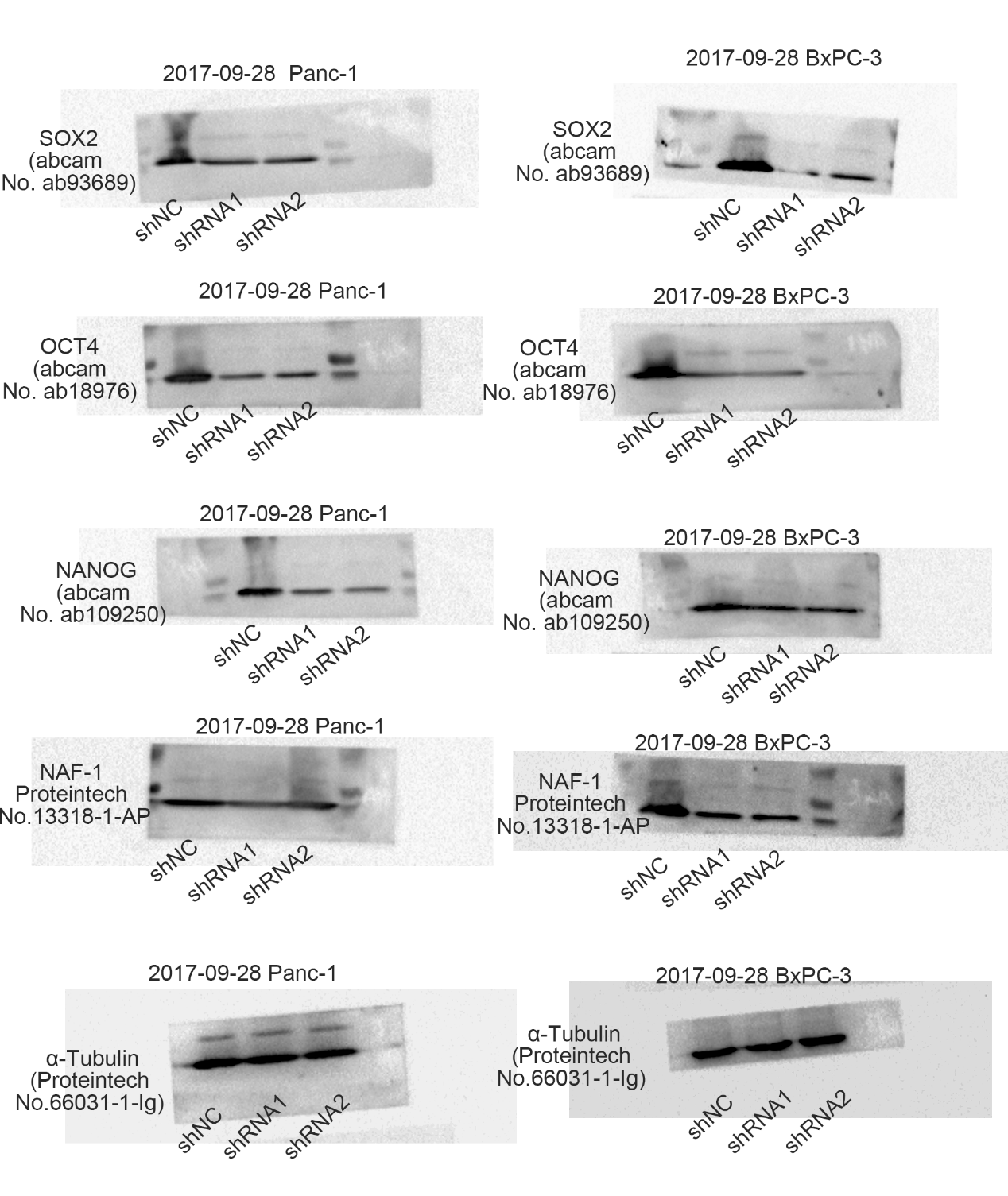


*This digit record is for Figure 7 that we repeated the* WB results*.*

*
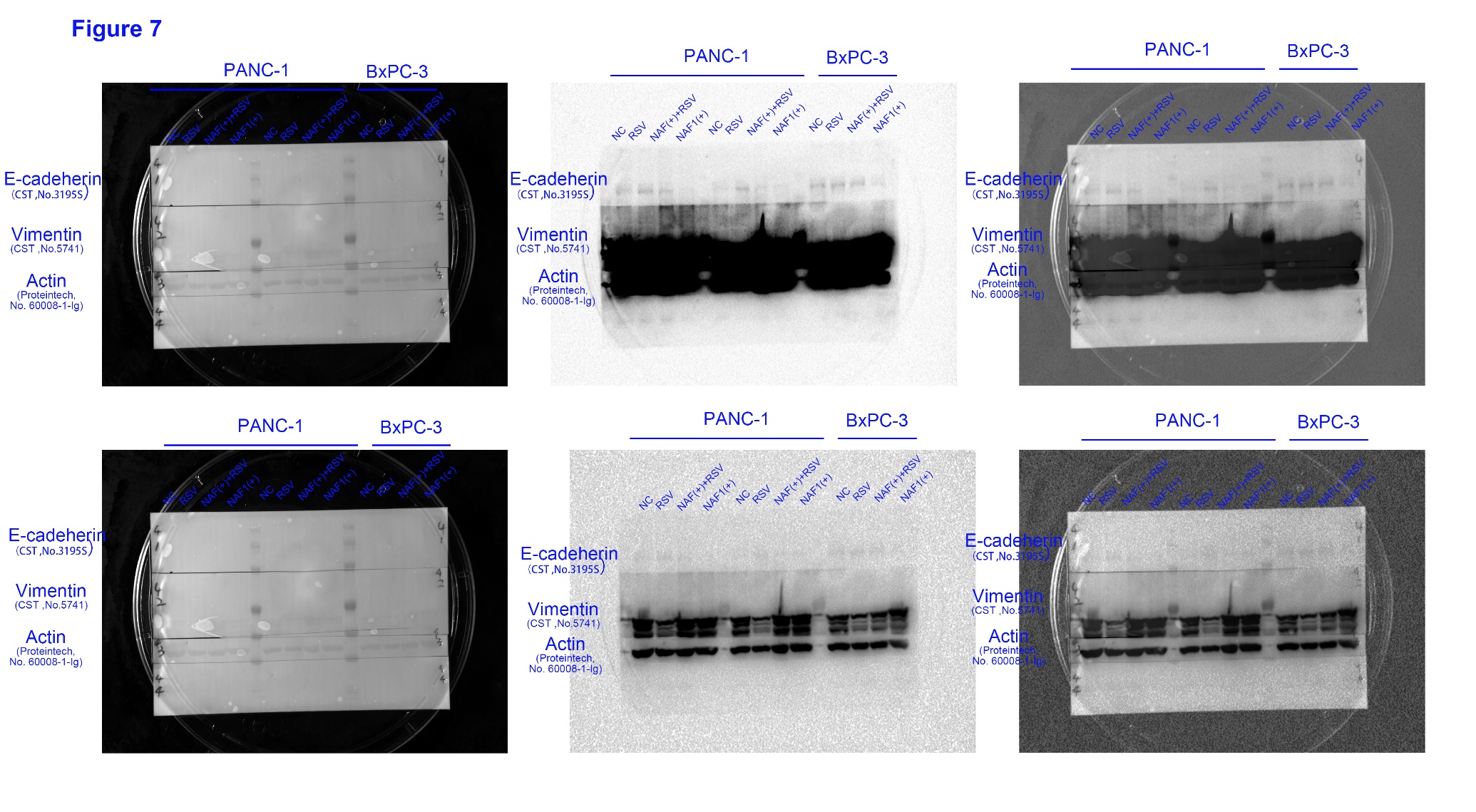
*
